# Raman Spectroscopy Reveals Photobiomodulation-Induced α-Helix to β-Sheet Transition in Tubulins: Potential Implications for Alzheimer’s and Other Neurodegenerative Diseases

**DOI:** 10.3390/nano14131093

**Published:** 2024-06-26

**Authors:** Elisabetta Di Gregorio, Michael Staelens, Nazanin Hosseinkhah, Mahroo Karimpoor, Janine Liburd, Lew Lim, Karthik Shankar, Jack A. Tuszyński

**Affiliations:** 1 Department of Physics, Faculty of Science, University of Alberta, Edmonton, AB T6G 2E1, Canada; 2 Department of Mechanical and Aerospace Engineering (DIMEAS), Faculty of Biomedical Engineering, Polytechnic University of Turin, 10129 Turin, Italy; 3 Department of Physics, Freie Universität Berlin, 14195 Berlin, Germany; 4 Instituto de Física Corpuscular, CSIC–Universitat de València, Carrer Catedràtic José Beltrán 2, 46980 Paterna, Spain; 5 Vielight Inc., Toronto, ON M4Y 2G8, Canada; 6 Department of Electrical and Computer Engineering, Faculty of Engineering, University of Alberta, Edmonton, AB T6G 1H9, Canada; 7 Department of Data Science and Engineering, Silesian University of Technology, 44-100 Gliwice, Poland

**Keywords:** proteins, protein dynamics, protein structure, non-invasive therapies, low-level laser therapy, spectroscopy, amide bands, amide I, spectral decomposition

## Abstract

In small clinical studies, the application of transcranial photobiomodulation (PBM), which typically delivers low-intensity near-infrared (NIR) to treat the brain, has led to some remarkable results in the treatment of dementia and several neurodegenerative diseases. However, despite the extensive literature detailing the mechanisms of action underlying PBM outcomes, the specific mechanisms affecting neurodegenerative diseases are not entirely clear. While large clinical trials are warranted to validate these findings, evidence of the mechanisms can explain and thus provide credible support for PBM as a potential treatment for these diseases. Tubulin and its polymerized state of microtubules have been known to play important roles in the pathology of Alzheimer’s and other neurodegenerative diseases. Thus, we investigated the effects of PBM on these cellular structures in the quest for insights into the underlying therapeutic mechanisms. In this study, we employed a Raman spectroscopic analysis of the amide I band of polymerized samples of tubulin exposed to pulsed low-intensity NIR radiation (810 nm, 10 Hz, 22.5 J/cm^2^ dose). Peaks in the Raman fingerprint region (300–1900 cm^−1^)—in particular, in the amide I band (1600–1700 cm^−1^)—were used to quantify the percentage of protein secondary structures. Under this band, hidden signals of C=O stretching, belonging to different structures, are superimposed, producing a complex signal as a result. An accurate decomposition of the amide I band is therefore required for the reliable analysis of the conformation of proteins, which we achieved through a straightforward method employing a Voigt profile. This approach was validated through secondary structure analyses of unexposed control samples, for which comparisons with other values available in the literature could be conducted. Subsequently, using this validated method, we present novel findings of statistically significant alterations in the secondary structures of polymerized NIR-exposed tubulin, characterized by a notable decrease in α-helix content and a concurrent increase in β-sheets compared to the control samples. This PBM-induced α-helix to β-sheet transition connects to reduced microtubule stability and the introduction of dynamism to allow for the remodeling and, consequently, refreshing of microtubule structures. This newly discovered mechanism could have implications for reducing the risks associated with brain aging, including neurodegenerative diseases like Alzheimer’s disease, through the introduction of an intervention following this transition.

## 1. Introduction

Healthy cellular function and structure are intrinsically linked to the integrity of tubulins. Tubulins are abundant, hydrophilic, and highly conserved cytoskeletal proteins found in all eukaryotic cells, which play a critical role in the structure and function of microtubules (MTs). Eukaryotic cells typically contain ∼3–4% tubulin [1,2,3,4,5,6]. Notably, however, mammalian brain tissue is particularly rich in tubulin content, consisting of ∼10% or more of the total protein content [1,3,4,5].

**Figure 1:**
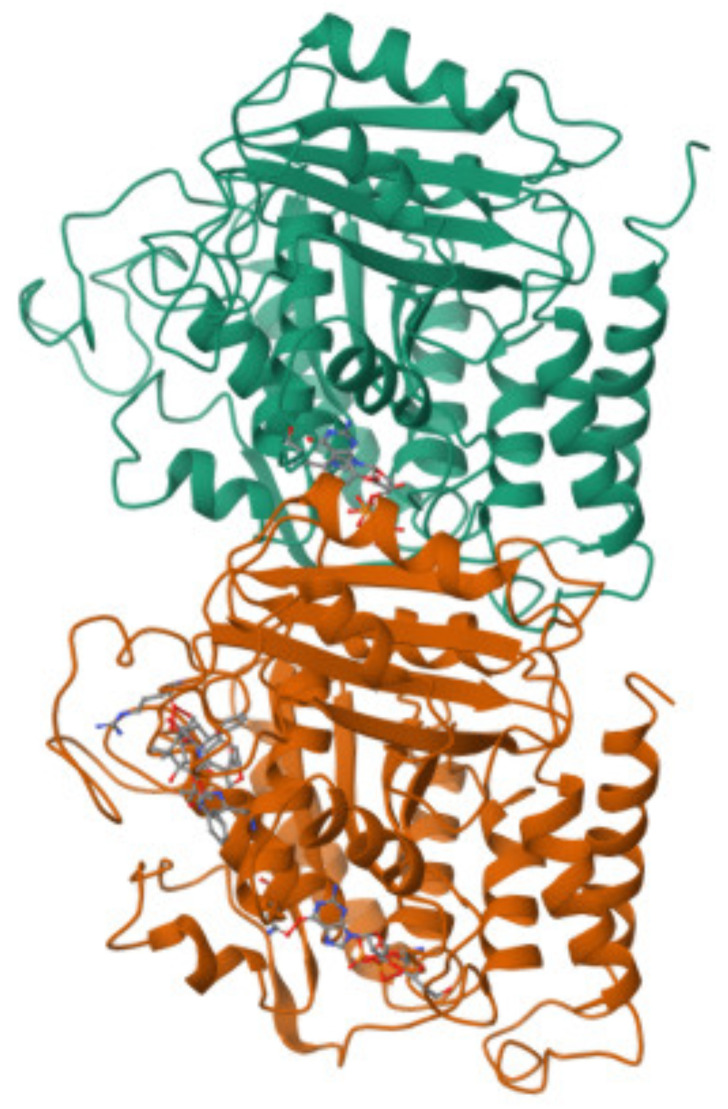
Experimental PDB structure of the αβ-tubulin heterodimer obtained by Nogales et al. at 3.7 Å resolution using electron crystallography (PDB ID: 1TUB) [10,12].

Tubulin has a heterodimeric structure composed of two closely related monomeric subunits, α- and β-tubulin, which combine via protein-folding and dimerization processes. Both monomers have molecular weights of ∼55 kDa each, share an amino acid sequence homology of ∼40–55% [7,8,9,10], and comprise a pair of β-sheets surrounded by α-helices [10]. Their secondary structure compositions are dominated by α-helices (which is generally true for globular proteins [11]). The three-dimensional structure of the αβ-tubulin heterodimer sourced from the Protein Data Bank (PDB; PDB ID: 1TUB) is shown in Figure 1 [10,12].

Regarding the functionality of tubulin, nucleation and the polymerization rate are attributed to α-helices, whereas β-sheets play a dual role in regulating these functions and contributing to the stability of this highly dynamic protein [13]. Additionally, αβ-tubulin heterodimers comprise oppositely charged ends, with the negative and positive ends formed by α-tubulin and β-tubulin, respectively. In contrast with its ordered structure, tubulin also presents a disordered portion: the negatively charged C-terminal tails, which play an important role in the interaction between tubulin and microtubule-associated proteins [14].

Members of the tubulin protein family are known to possess unique electrostatic properties [15] that are fundamental to their ability to form MTs. αβ-tubulin dimers polymerize head to tail into intrinsically polar linear protofilaments that can further assemble into metastable MTs through lateral tubulin–tubulin interactions; generally, MTs comprise 13 protofilaments arranged in a tubular lattice configuration. MTs are dynamic structures that play crucial roles in many cellular processes, such as cell division and chromosome segregation [16,17]; cell movement and motility [18,19]; maintaining cell structure and rigidity [20]; and the transport of vesicles and organelles via kinesin and dynein motor proteins [21].

In the formation of MTs, αβ-tubulin binds guanosine triphosphate (GTP) at two different binding sites, one exchangeable (in β-tubulin) and one non-exchangeable (in α-tubulin). Hydrolyzation of GTP at the exchangeable site allows tubulin assembly [10,22,23] into a mainly GDP-tubulin microtubule, with a final portion of GTP-bound tubulin known as the GTP cap. It is the presence of this cap that makes MT polymerization possible [23,24]. When this piece of MTs is lost, the catastrophe phenomenon occurs, causing MTs to shrink instead of grow [24]. Growth will only resume after the GTP cap is reacquired. This process is known as rescue [25,26]. Thus, MTs have dynamic behavior, alternating between phases of shrinking and growing, permitting them to be reshaped in cells. This particular characteristic, which is known as dynamic instability [24,27], is pivotal for MT integrity and, if lost, can alter cell division properties. In healthy cells, time and space are important factors in the regulation of MT dynamics, even across the cytoplasm [28]. In particular, during mitosis, interphase MTs disassemble to form the mitotic spindles, which are about 100 times faster at assembling/disassembling [29]. The mitotic spindle is responsible for chromosome segregation. After the cell is completely divided, MTs forming the mitotic spindle reassemble into cytoplasmic MTs [29].

MTs possess a variety of interesting and distinct electrical properties (reviewed in detail in Ref. [30]), such as electrical conductance and impedance [31], as well as a highly negatively charged surface due to the large negative electrostatic charge of αβ-tubulin dimers ($Q_{\mathrm{eff}}∼-23\;e$ for a dimer in an MT [32]); thus, they have been considered as bionanowires that, in addition to supporting ionic transport [33,34], could be responsible for intracellular signaling [35,36,37,38]. Given these unique electrical properties and the highly polar nature of MTs, they have been considered a potential target for electromagnetic field (EMF)-based therapies. Numerous investigations, under specific conditions and parameters, have documented diverse impacts of EMFs on MTs in solution, such as the alignment of MTs in the presence of electric fields [32,39,40,41,42,43], the disassembly of MTs by intense terahertz pulses [44], and effects on MT polymerization induced by low-intensity near-infrared (NIR) light [45].

In neurons, MTs are responsible for the maintenance of neuron shape and structure, neuronal soma migration [46], the growth and structure of axons [47], protein transport in axons and dendrites [48], and the support of morphological changes in dendrites potentially associated with neuroplasticity [46]. MTs in brain cells may exhibit varying levels of stability compared to other cell types, depending on the specific context and cellular functions they are involved in; however, on average, MTs are more stable in neurons compared to other cells [46]. Additionally, in axons and dendrites, neuronal MTs are found in unique and curious configurations as uniform parallel aligned arrays [46,49]. A plethora of studies have reported MT loss and dysfunction in connection with the onset and progression of neurodegenerative diseases (NDs), such as Alzheimer’s disease (AD) [50,51,52,53,54,55,56,57].

Photobiomodulation (PBM) uses low-intensity, non-thermal, and non-ionizing sources of electromagnetic (EM) radiation, typically in the visible red and NIR regions of the EM frequency spectrum, to induce positive physiological changes and health outcomes. Several small clinical studies of PBM for NDs have demonstrated remarkable results [58,59,60,61]. For example, statistically significant improvements in patients’ Alzheimer’s Disease Assessment Scale–Cognitive Subscale (ADAS-Cog [62]) scores were reported in two studies employing 12-week transcranial–intranasal 810 nm PBM (−6.73 points vs. baseline after 12 weeks, p<0.023 [58]; −5.2 points vs. baseline after 12 weeks, p=0.007 [59]). Notably, both of these small studies observed mean improvements in ADAS-Cog scores that were markedly greater than those reported in a phase III clinical trial involving 10 mg/day donepezil therapy (∼−2 points vs. baseline after 12 weeks, p<0.0001) [63], which for a long time has been the standard of treatment for AD. Moreover, with PBM, patients with mild-to-moderately severe dementia experienced noteworthy enhancements, including improved sleep, reduced anxiety, and increased functional ability, without any negative adverse effects [58].

Additionally, the neuroprotective effects of PBM for AD have been demonstrated both in vivo [64,65,66,67,68,69] and in vitro [70]. This has led to an increased interest in such therapies, and the number of studies on their efficacy in treating NDs has seen a substantial increase (see Ref. [71] for a review); several clinical trials for treating NDs are currently ongoing [72,73,74]. Despite the promises, the literature recognizes that the mechanisms of action underlying the observed efficacy of PBM are still not entirely clear, and studies that will help us to understand the biophysical and subcellular effects at the molecular level are lacking [75]. Thus, more research on the molecular and biophysical mechanisms of action is highly warranted. Findings in this area would contribute to explaining the mechanisms underlying the effect of PBM on the pathophysiology of ND conditions such as Alzheimer’s disease. In this work, we present the results of such a study aimed at investigating the effects of the pulsed NIR light employed in PBM therapy on the secondary structures of tubulin and MTs—fundamental components of the cytoskeleton in eukaryotic cells. It involves a significant transition of α-helical to β-sheet arrangements.

We used Raman spectroscopy to compare the secondary structure compositions of polymerized tubulin in buffer solutions with and without exposure to pulsed NIR light. Raman spectroscopy [76,77] and other spectroscopic techniques, such as Fourier-transform infrared (FTIR) spectroscopy [78,79] and far-ultraviolet (UV) circular dichroism (CD) spectroscopy [80,81], are typically employed to study the secondary structures of proteins. Raman spectroscopy is a scattering-based technique that measures the inelastic scattering of light by molecules, which has several advantages over other spectroscopic techniques. One of the advantages of Raman spectroscopy over infrared spectroscopy is that $H_{2}O$ vibrations have less influence on Raman spectra, eliminating the need for $D_{2}O$ and reducing the error related to background subtractions [76,82]. Additionally, Raman spectroscopy is more feasible for studying turbid solutions, such as solutions of polymerized tubulin, whereas methods such as CD are not suitable due to potential distortion in the measured signal caused by the turbidity.

In summary, Raman spectroscopy is a powerful, non-destructive, label-free method that has demonstrated utility for the chemical analysis of biological and non-biological samples. With this technique, we could observe macromolecule conformation modifications, which translate to shifts in the frequency bands acquired through this methodology [76]. Three Raman bands, amide I, II, and III (1600–1700 cm^−1^ [83], 1510–1580 cm^−1^ [84], and 1220–1310 cm^−1^ [85], respectively), are particularly useful for evaluating proteins and peptide structures [86]. C=O stretching vibrations account for around 80% of the amide I band [83]. The remainder is related to C–N out-of-plane stretching [83]. In contrast, the amide II band is less sensitive to alterations in protein conformation [84,87]. It accounts primarily for in-plane bending of N–H groups (40–60%) and vibrations related to the stretching of the C–N groups (18–40%) [83,84,87], while C=O bending and C–C stretching have little influence on this band [83]. Finally, amide III peaks are related to the bending of in-plane N–H groups and C–N stretching [85].

Only a couple of studies have performed Raman spectroscopic analyses of the secondary structures of tubulin [13] and MTs [13,88]. To validate our methodology, we compared our Raman spectroscopy results for unexposed tubulins with those from various other studies in the literature that utilized Raman spectroscopy, CD, and FTIR, ensuring the consistency of our technique’s outcomes. Subsequently, we exploited our validated method to determine how tubulin changes its internal structure when exposed to pulsed low-intensity NIR light. As far as we are aware, this is the first such study to report changes in the secondary structures of tubulin induced by NIR radiation.

Beyond the implications of this study regarding PBM therapy for NDs, our results could have broader applications in the field of nanotechnology. MTs are a nanoscale protein polymer with multifunctional characteristics, some of which have been investigated from the technological point of view before but not in terms of specific structural changes to the building block tubulin when exposed to NIR radiation. This could provide a control in the processes in which MTs could be used, for example, in mass transport using motor proteins as molecular machines and MTs as an architecture of roadways for these molecular motors. Nanotechnological applications of this work are anticipated in the area of biosensors, bioelectronics, and biological computing using MTs and their interconnections as EM-controllable signal processing units in complex circuits.

The remainder of this article is organized as follows. In Section 2, we briefly describe the materials, equipment, and methodology employed in this study. The results are presented in Section 3, beginning with a comparison of our results obtained for the secondary structure composition of unexposed tubulin (control samples) with values obtained in several other studies in the literature both to validate our methodology and to resolve some of the tension between the different values reported by these studies. Thereafter, we present our results for the secondary structures of the NIR-exposed tubulin samples and contrast these results against those obtained for the unexposed samples. We discuss these results and present several ensuing hypotheses regarding their possible connection to the reported efficacy of PBM for treating AD in Section 4. Lastly, conclusions and future outlooks are provided in Section 5.

## 2. Experimental Methodology

### 2.1. Reconstitution of Tubulin Samples

Unlabeled, ultra-pure tubulin derived from porcine brain, purchased from Cytoskeleton, Inc. (Denver, CO, USA; T240), was employed in the following experiments. T240 samples were stored at 4 °C and later resuspended to 2.5 mg/mL tubulin. This was achieved by adding 360 µL of ice-cold G-PEM buffer (GTP-supplemented PEM buffer: 80 mM PIPES pH 6.9, 0.5 mM EGTA, and 2 mM $\mathrm{Mg}{\mathrm{Cl}}_{2}$) and 40 µL of Microtubule Cushion Buffer (PEM buffer in 60% *v*/*v* glycerol) to each vial, and then placing the protein samples on ice. The concentration of tubulin employed in our experiments (∼22.7 µM) is consistent with cytoplasmic concentrations found in living cells [89,90]. The G-PEM buffer was prepared by adding 990 µL of PEM buffer to 10 µL of 100 mM GTP; thus, the final GTP concentration of the G-PEM buffer was 1 mM. After reconstitution, the samples were aliquoted into experimental amounts of 1 mL and snap-frozen through immersion in liquid nitrogen to avoid protein denaturation. Finally, the samples were stored at −80 °C until they were used in the experiments.

### 2.2. Near-Infrared Exposure of Tubulin

The exposure of reconstituted tubulin samples was performed with the intranasal LED applicator of the Vielight Neuro Alpha transcranial–intranasal brain PBM device; its parameters are reported in Table 1. These specific parameters have demonstrated efficacy in treating dementia [58] and traumatic brain injury [91] with transcranial–intranasal PBM. Additionally, a recent randomized sham-controlled crossover study utilizing transcranial–intranasal PBM with the same parameters reported significant modulation of neural oscillations observed in resting-state electroencephalography of healthy volunteers [92]. In their scenario employing the intranasal LED only, significant enhancements in beta and gamma waves were observed 10 and 30 min after treatment [92]. Moreover, on its own, the intranasal LED applicator has shown potential as a treatment method for neurological disorders [93,94].

Tubulin samples collected from the −80 °C freezer were exposed for 30 min inside a 4 °C fridge to prevent polymerization of the samples during exposure. To avoid movement of the sample with respect to the LED, it was fixed directly to the intranasal applicator and then to the inside of a cardboard cryo box. The box was also utilized to keep the sample isolated and in the dark during exposure to avoid light diffusion and reflection. From the power density of the LED, we can calculate both the delivered energy and the approximate strength of the electric field generated. For a 30 min exposure, the total energy delivered amounts of 22.5 J (i.e., a net dose of 22.5 J/cm^2^); the electric field strength is approximately 4.3 V/cm. Separate tubulin samples that were not subjected to any NIR exposure were preserved for use as control samples. After exposure and prior to performing Raman spectroscopy, the tubulin samples were polymerized into MTs by placing them in a 37 °C incubator for 60 min. Two independent experiments and subsequent measurements were performed.

### 2.3. Raman Spectroscopy

A 5 µL droplet of polymerized tubulin solution, either untreated or NIR-exposed, was deposited onto a glass slide for measurement. Raman spectra were acquired at room temperature with a 532 nm laser operating at 50 mW power, a 1200 lines per mm grating, and an exposure time of 1 s, using a Renishaw inVia™ Qontor^®^ confocal Raman microscope (Renishaw Ltd., Mississauga, ON, Canada). This instrument offers high spatial resolution (better than 300 nm for 532 nm excitation) and is particularly well-suited for the study of polymers. The resulting spectra reported here derive from at least two samples measured. Several points of the same sample—focused through the 50× objective—were measured, capturing multiple acquisitions (4–5) for each position. The multiple acquisitions obtained were automatically averaged by Renishaw’s WiRE™ software (v. 5.3), which manages the collection of Raman data.

### 2.4. Data Processing and Spectral Decomposition

The data were processed using Matlab^®^ R2022a (v. 9.12). After the acquisition of spectra, range reductions (to 350–2700 cm^−1^) and baseline corrections were implemented. Asymmetric least-squares smoothing with a threshold of 0.01, a smoothing factor of 5, and 10 iterations was employed for the baseline corrections [95]. The data were smoothed using Savitzky–Golay filtering [96], available in the Matlab^®^ Signal Processing Toolbox, based on a second-order polynomial and with a 17-point window. Additional measurements were conducted for both the blank glass slide and the buffer solution, and in both cases, the resulting spectra in the amide I region exhibited no band structure contributions, eliminating the need for any background subtractions associated with these potential contributions. For spectral deconvolution, all the measured spectra were restricted to the amide I band and normalized between 0 and 1. *Z*-score normalization was chosen to highlight relative differences in spectral characteristics across samples, minimizing the influence of absolute intensity levels and ensuring that observed variations reflect differences in sample composition or structure rather than experimental conditions or instrument settings. Peak finding was performed by analyzing the second derivative of the spectra. Previous research has shown that the biophysics of protein-folding processes, of which secondary structures are a consequence, can be effectively described by a Voigt profile [97]. Accordingly, peak deconvolution of the measured Raman amide I spectra was performed with a Voigt profile distribution, which is defined as a convolution of Lorentzian and Gaussian distributions:

(1) $$\begin{array}{c} f_{L}\left(x\right)=\frac{2A}{\pi }\frac{w_{L}}{4(x-x_{c}{)}^{2}+w_{L}^{2}}, \end{array}$$

(2) $$\begin{array}{c} f_{G}\left(x\right)=\sqrt{\frac{4\ln2}{\pi w_{G}^{2}}}\;\exp\left(-\frac{4\ln2}{w_{G}^{2}}x^{2}\right), \end{array}$$

where *A* represents the area, $x_{c}$ represents the center, and $w_{L}$ and $w_{G}$ are parameters specifying the Lorentzian and Gaussian full width at half maximum, respectively. Explicitly, it can then be written as [98]

$$\begin{array}{c} y\left(x\right)=y_{0}+f_{L}\left(x\right)*f_{G}\left(x\right) \end{array}$$

(3) $$\begin{array}{cc} & =y_{0}+A\frac{2\ln2}{\pi^{3/2}}\frac{w_{L}}{w_{G}^{2}}\times \int_{-\infty }^{\infty }\left(\frac{e^{-t^{2}}}{{\left(\sqrt{\ln2}\frac{w_{L}}{w_{G}}\right)}^{2}+{\left(2\sqrt{\ln2}\frac{x-x_{c}}{w_{G}}-t\right)}^{2}}\right)\;dt. \end{array}$$

Curve fitting was performed using the Levenberg–Marquardt minimization algorithm function available in Matlab^®^ [99,100]. The coefficient of determination, $R^{2}$, was used to evaluate the goodness of the fits. We considered values for this parameter optimal when they exceeded 0.99. Following the fitting procedure, the amide I vibrational peak areas were used to evaluate the secondary structure content of each protein sample. This analysis was performed by adding the areas of all the amide I peaks obtained that contribute to the secondary structures and calculating the individual contribution of each peak associated with a particular secondary structure: α-helices, β-sheets, random coils, and β-turns [84,101]. This is based on the assumption that the Raman cross-section for the mentioned structures is the same, as discussed by Surewicz et al. [102] and Sane et al. [103]. The peaks associated with each secondary structure were assigned to the specific sub-bands of the amide I envelope summarized in Table 2 [104]; however, there are no universally agreed-upon definitions for these characteristic bands, and hence, the particular values stated differ slightly throughout the literature. Following these assignments, the peaks obtained from the deconvolution of the processed spectra were compared against the normalized raw data to ensure that no processing-related artifacts affected the quantification of secondary structures; no obvious artifacts influencing the results were identified. Finally, the mean percentages of secondary structures were calculated for both groups of tubulin samples studied.

### 2.5. Statistical Analyses

Statistical hypothesis testing was performed to compare the resulting mean secondary structure compositions of the unexposed tubulin samples with other results reported in the literature, as well as with the results obtained for the NIR-exposed tubulin. Under a normality assumption, statistically significant differences between results were established using Welch’s unequal variances *t*-tests [105,106] with a significance level of α=0.05. All statistical analyses were performed using Matlab^®^ R2023a (v. 9.14).

## 3. Results

We first report the results of our Raman spectroscopic analyses of the secondary structure compositions of the unexposed control samples. These results are compared with those from other studies available in the literature that analyzed the conformation of tubulin and microtubules. This is followed by a presentation of our secondary structure results obtained for the NIR-exposed tubulin samples and how they compare to the results for the control samples.

### 3.1. Raman Spectra and Secondary Structures of Polymerized Unexposed Tubulin

**Figure 2:**
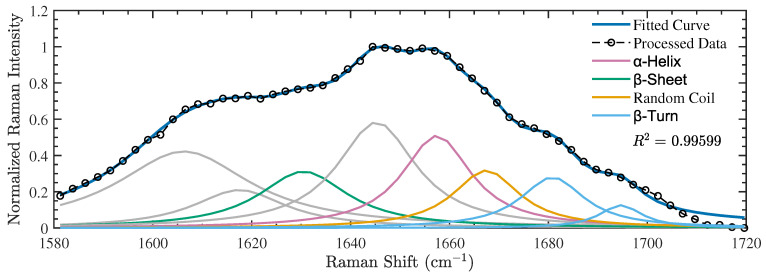
A representative example of one of the amide I Raman spectra obtained for the control (unexposed) polymerized tubulin samples (labeled as Control 1 in Table 3). Gray curves represent peaks obtained from the spectral deconvolution that are unassociated with any secondary structures.

The secondary structure compositions obtained for our control samples in the two independent experiments performed are presented in Table 3. A sample of one of the control spectra obtained in these experiments and the resulting spectral deconvolution of the amide I band is shown in Figure 2 (the remaining spectra are available in the Supplementary Material file, Figures S1–S3). As previously stated, each peak was assigned to a characteristic secondary structure to estimate its total percentage. In both experiments, we find that the secondary structure compositions of the unexposed samples are dominated by α-helices, which is typically the case for globular proteins. In two of the three measurements, the results indicate that the second-most abundant structures are β-sheets. The average (±SD) results, reported in the first row of Table 4, are consistent with this trend. In particular, we find an average secondary structure composition dominated by 36.0±4.2% α-helices followed by 26.7±7.1% β-sheets.

Two studies that analyzed the conformation of polymerized tubulin are available in the literature. Both studies employed Raman spectroscopy and subsequent deconvolution of the amide I band [13,88]. Their average results are presented in the upper portion of Table 4 (uncertainties are also displayed for every study that explicitly reported such values). Welch’s unequal variances *t*-tests indicate that our results differ significantly from those reported by Audenaert et al. [13] for all secondary structures analyzed except β-turns. Notably, however, we find good overall agreement with the results obtained by Simić-Krstić et al. [88], with no significant differences obtained for any of the structures analyzed, suggesting that our techniques are consistent. Moreover, for both α-helices and β-sheets, we find excellent agreement with their results within one error interval.

Several other studies in the literature have analyzed the conformation of tubulin dimers using different spectroscopic techniques. We compared our results with three such studies: Ventilla et al. [107], which employed far-UV CD spectroscopy; de Pereda et al. [108], which included two independent analyses using both far-UV CD spectroscopy and FTIR spectroscopy; and finally, Afrasiabi et al. [109], which also utilized far-UV CD spectroscopy. Their results on the secondary structure composition of tubulin dimers are presented in the lower portion of Table 4. We find agreement with the results of both analyses reported by de Pereda et al. [108] for all secondary structures, as well as with those reported in the study by Afrasiabi et al. [109] for α-helices and β-sheets. The discrepancy between our average result for random coil structures and that reported by Afrasiabi et al. appears to be due to their analysis not separately quantifying β-turns; integrating our β-turn and random coil results yields no statistically significant difference compared with their random coil result (p=0.0972). On the other hand, our results largely disagree with those reported by Ventilla et al. [107].

In summary, for the main secondary structures—α-helices and β-sheets—we find the best agreement with the values reported by de Pereda et al. (FTIR, p=0.717) and Simić-Krstić et al. (Raman, p=0.943), respectively. Our comparative analysis with the literature suggests that our approach using Raman spectroscopy was consistent with other techniques. Therefore, our results for the secondary structure composition of the unexposed tubulins are credible as baselines for comparing the effects of NIR irradiation. Detailed results from all these statistical tests can be found in Section S2 of the Supplementary Material, Tables S1–S6. Relevant information on the different experiments and analyses performed by each group we made comparisons with is also provided therein (see Table S7 for a comparative overview). Although it is not entirely clear what particular factors might be responsible for some of the observed disagreements between results, these experimental and methodological differences are notable and likely account for some of the variances.

### 3.2. Raman Spectra and Secondary Structures of Polymerized NIR-Exposed Tubulin

**Figure 3:**
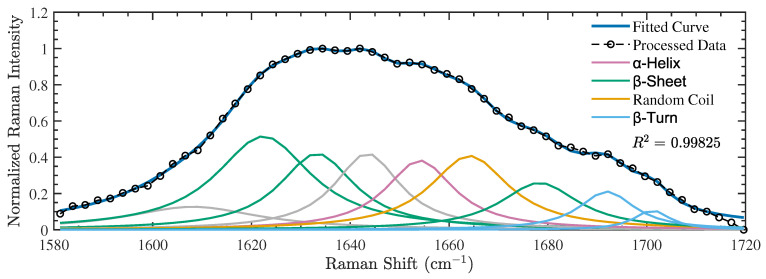
A representative example of one of the amide I Raman spectra obtained for the NIR-exposed polymerized tubulin samples (labeled as Exposed 1 in Table 5). Gray curves represent peaks obtained from the spectral deconvolution that are unassociated with any secondary structures.

We now turn to our results for the secondary structure compositions of polymerized NIR-exposed tubulin obtained by deconvoluting their measured Raman spectra using the same procedure applied to the control samples. The secondary structure compositions derived from the exposed samples in the two independent experiments are detailed in Table 5. Figure 3 presents an example of one of the exposed spectra gathered during these experiments, along with the consequent spectral deconvolution of the amide I band (additional spectra can be found in the Supplementary Material, Figures S4–S6). Notably, our results for the three different NIR-exposed samples indicate a significant change in conformation, in which the β-sheets dominate the secondary structure composition. The average results (±SD) are reported in Table 6 alongside the mean results obtained for the control group and the statistical test results.

Compared to the unexposed samples, marked differences can be observed: the NIR-exposed tubulin samples present average α-helix and β-sheet contents of 13.9±1.0% and 54.3±7.7%, respectively. The results of Welch’s unequal variances *t*-tests indicate statistically significant differences in the mean values of β-sheets (p=0.0104) and α-helices (p=0.00887). The differences in β-turn and random coil (undefined) structures were not found to be statistically significant. The complete results of these statistical tests can be found in Table S8 of the Supplementary Material. In conclusion, we found that in vitro exposure of tubulin to pulsed low-level NIR radiation led to a highly statistically significant reduction in α-helices and a concurrent statistically significant increase in β-sheets.

## 4. Discussion

The Raman spectroscopy results of this study appear to indicate that low-intensity, non-ionizing NIR radiation interacts with tubulin at the molecular level, modifying its secondary structures, as evidenced by significant changes in the amide I band. This particular Raman mode is highly responsive to alterations in the hydrogen bonding strength between N–H and C=O groups [83]. In this region, the Raman spectra of the NIR-exposed and subsequently polymerized tubulin samples illustrate the transformation from a conformation dominated by α-helical content to one dominated by β-sheets. Gautam et al. [85], in their Raman spectroscopic study of various gene mutants, accounted for such a loss in α-helical content in other proteins as “structural unfolding and/or denaturation” (where the mechanism underlying unfolding is likely the breaking of weak H-bonds [110]). At the same time, they justified β-sheet formation as the result of the interaction between exposed hydrophobic residues of different molecules with each other [85].

Another example of the same protein unfolding–refolding behavior is reported by Perillo et al. [111]. In their work concerning the impact of mechanical forces on protein structures, they witnessed a reduced Amide I band signal intensity and a decrease in α-helix content in response to the applied strain forces [111]. These effects were consistent with results obtained in a similar prior study conducted by the same authors [112]. Independently, this behavior had also been previously demonstrated in another Raman spectroscopy study, reporting an α-helix to β-sheet transition in the proteins of strained keratin fibers, evidenced by a progressive increase in β-sheet content and decrease in α-helices as a function of the applied strain intensity [113].

Various studies in the literature also highlight interesting ways in which electromagnetic stimuli can induce conformational changes in proteins [109,114,115,116,117,118,119]. For instance, using X-ray crystallography, Lundholm et al. observed terahertz-radiation-induced (0.4 THz, 62 mW/cm^2^) steady-state secondary structure changes in lysozymes characterized by α-helix compression, which the authors attributed to resonant interactions [116]. In another study, which investigated the effects of extremely low-frequency magnetic fields (−2.4–2.4 mT, 50 Hz, 5.0 min exposure) on cAMP response element-binding protein (CREB), FTIR spectroscopic analysis revealed lasting conformational changes evidenced by varying spectral band shifts in the amide II, IV, and VI regions [118].

Perhaps most notably in comparison to our findings, Bekard and Dunstan [115] reported that low-intensity oscillating electric fields (EFs) affected the structures of bovine serum albumin and lysozyme in solution. In particular, deconvolution of far-UV CD spectra obtained from room-temperature lysozyme solutions exposed for 3 h to oscillating EFs (3 V/cm, 10 Hz) revealed a transition from a secondary structure dominated by α-helices before exposure (31% α-helices, 20% β-strands) to one with a higher fraction of β-strands post-exposure (19% α-helices, 28% β-strands) [115]. The authors attributed this to protein unfolding caused by the disruption of H-bonds by frictional energy dissipation from the EF-induced electrophoretic motion of the proteins [115]. Similarly, in our study, it is possible to observe an analogous transition in the secondary structure of NIR-exposed tubulin samples (810 nm, 10 Hz, ∼4.3 V/cm). Furthermore, our findings, based on non-simultaneous NIR exposure and acquisition of spectra, also indicate potential long-term effects or extended relaxation times. Interestingly, in a recent in vivo study on the effects of transcranial PBM on human subjects, significant changes in EEG were observed both 10 and 30 min after treatment (using the same device employed in this study) [92].

While there has yet to be any other study in the literature investigating the effects of pulsed NIR light on the secondary structures of tubulin or MTs, we can draw comparisons with a study by Afrasiabi et al. [109], which, to the best of our knowledge, appears to be the most closely related one. Specifically, these researchers performed far-UV CD spectroscopy on tubulin dimers (2 mg/mL) subjected to 30 min in vitro exposure to extremely low-frequency electromagnetic fields (ELF-EMFs) with frequencies of 50, 100, and 217 Hz and an intensity of 0.2 mT [109]. Consistent with our results for NIR-exposed tubulin with the same exposure time and a similar concentration (2.5 mg/mL), their corresponding secondary structure analyses of CD data revealed a reduction in α-helices and an increase in β-sheets for all three frequencies of ELF-EMFs studied [109]. In addition to their CD spectroscopy analyses, several other techniques were employed, including transmission electron microscopy and turbidity assays, to study MT polymerization. Results from both methods displayed a reduction in polymerization and an increase in the nucleation time (“lag” phase) observed for the exposed tubulin samples for each of the ELF-EMFs studied, which the authors associated with the secondary structure alterations induced by exposure [109].

These findings are consistent with the results of our previous study [45], in which we also employed turbidity measurements to explore how exposing tubulin to the same PBM device used in this study affects its polymerization into MTs. For the same tubulin concentration and exposure conditions used in this study, we obtained a reduction in the polymerization rate and the final polymer mass of the exposed tubulin samples compared to the control samples, as well as an increase in the time required to produce 10% of the maximal value of polymer (i.e., an increased nucleation phase) [45]. This reduction in the polymerization rate is a key factor in determining if MTs will continue to grow or start to shrink. In particular, if the addition of new GTP-bound tubulin molecules occurs faster than the rate of GTP hydrolysis, the GTP cap is maintained and MT growth will continue; conversely, if the polymerization rate falls below that of GTP hydrolysis, the tubulin–GTP subunit at the growing end of the MT will undergo hydrolysis, leading to the catastrophe phenomenon [120]. Thus, the reduced rate of polymerization observed in the NIR-exposed tubulin samples could pose consequences for the stability of the ensuing MTs.

Additionally, in the Raman spectroscopic study of free and polymerized tubulin conducted by Audenaert et al., the GTP- and GDP-bound states of tubulin dimers were distinguished by a significant decrease in ordered α-helices and a concurrent increase in antiparallel β-sheets observed in the latter state [13]. In connection with this study, this suggests the possibility that the MTs assembled from NIR-exposed tubulin in our study might contain a larger proportion of GDP-bound tubulin in the MT lattice, again leading to increased MT instability.

These studies indicate a plausible relationship between NIR-exposure-induced changes in secondary structures and tubulin/MT polymerization dynamics. Based on these results, we present several hypotheses regarding this connection. First, we note that the lower final MT polymer mass measured for the NIR-exposed tubulin samples in our previous study can be interpreted through two distinct processes, which could happen concurrently: (1) reduced polymerization compared to the control samples, resulting from the increased nucleation time and decreased polymerization rate observed; and (2) reduced stability of the MTs assembled from NIR-exposed tubulin, leading to increased MT disassembly and hence a lower final polymer mass.

Likewise, in line with the first interpretation, we propose that the NIR-induced changes in secondary structural elements—in particular, the reduction in α-helices—cause a reduced polymerization rate and hindrance of nucleation that ultimately affect MT growth dynamics. Alternatively, in accordance with the second interpretation, we hypothesize that the induced conformational changes lead to reduced MT stability. A third likely possibility is that all of these effects occur simultaneously. This latter possibility is consistent with the literature regarding the role of α-helices in the functionality of tubulin. Notably, α-helices are believed to be involved in nucleation, tubulin–tubulin interactions along protofilaments [10,121], and lateral inter-protofilament interactions [121,122]. Table 1 in Ref. [121], based on data from Refs. [122,123], provides a comprehensive overview of the structural elements in tubulin, including α-helices and β-sheets, participating in the longitudinal and lateral interactions along and between protofilaments, respectively. The insights provided therein suggest that lateral interactions—which are of utmost importance in maintaining MT stability—predominantly involve α-helices, supporting our hypothesis that a reduction in such secondary structures could promote MT instability. Either way, our results have implications regarding the mechanisms underlying the efficacy of PBM in treating NDs such as AD.

In a recent study by Peris et al. [57], both ex vivo and in vivo experiments demonstrated that a characteristic aspect of MT dysfunction in early AD is that they become overly stable, which hinders neuronal activity. The authors initially demonstrated this through postmortem analyses of brain samples from AD patients, which exhibited increased levels of detyrosinated tubulin compared to samples collected from individuals without the disease [57]. Over time, the C-terminal end of α-tubulin in MTs naturally undergoes detyrosination; thus, high levels of tyrosinated tubulin are typically found in young, dynamic MTs, whereas detyrosinated tubulin is representative of aged, long-lived MTs. Further experiments performed using a heterozygous mouse model subjected to inhibited MT rejuvenation via downregulation of the tubulin tyrosine ligase gene—resulting in mice with an increased proportion of aged neuronal MTs—revealed consequential memory deficiencies and reduced synaptic content [57].

Similarly, in the study by Muhia et al., neurons from memory- and learning-impaired mice with an inactivated kinesin family member 21B gene (responsible for encoding a kinesin motor protein involved in synaptic vesicle transport along neuronal MTs) also displayed evidence of impaired microtubule dynamics [52]. Together, the results of these studies suggest that while a certain amount of stable MTs are needed for cellular support, a significant proportion of dynamic MTs is also necessary for proper brain function, and a disruption in this equilibrium is detrimental. In fact, for the brain to encode new memories, a precise balance between dynamic and stable microtubules in neurons appears to be foundational. Thus, we hypothesize that through NIR PBM therapy employing the parameters studied herein to treat AD patients, where this balance is disrupted, induced changes in tubulin secondary structures leading to altered polymerization dynamics and reduced MT stability could promote MT depolymerization and encourage cytoskeletal remodeling by enabling the replacement of old, overly stable MTs with new dynamic MTs.

Typically, a pronounced increase in β-sheets would be cause for concern, as it might exacerbate the pathogenesis of NDs. This is grounded in the understanding that, in many functional proteins, the conversion of normal α-helix structures into β-sheets is often linked to protein misfolding and aggregation connected with the formation of amyloids [124]. Tubulins, however, have not been traditionally associated with amyloid formation, nor are they generally known for their propensity to form amyloids; thus, they are not currently recognized as typical amyloid-forming proteins. This seems to mitigate the risks of misfolding and aggregation. In this context, the highlighted observations from our previous study [45] regarding PBM-induced alterations in tubulin dynamics and delayed polymerization become particularly significant. The notable positive clinical outcomes from PBM associated with the parameters deployed in this study suggest that these altered dynamics might contribute to the remodeling and renewal of the MT structures. Such an effect might be analogous to counteracting brain aging, offering potential benefits against AD and other NDs.

The rejuvenation of MTs could also have therapeutic applications regarding the neurovascular unit (NVU) [125,126]. The NVU—a critical functional unit of the brain comprising neurons, glia, and vascular cells—has been considered a potential therapeutic target for NDs [127,128]. In NDs, the NVU exhibits dysfunction [129]; some examples include reduced nitric oxide (NO) production in endothelial cells, pericyte dysfunction (e.g., loss of pericytes and impaired signaling), reduced cerebral blood flow, mitochondrial dysfunction (e.g., impaired adenosine triphosphate (ATP) production), and aberrant cell–cell signaling [130]. While several well-understood effects of NIR PBM therapy, such as increased mitochondrial ATP production [131,132] and NO release [133], can contribute significantly to restoring the function of the NVU, this study’s findings suggest additional potential mechanisms for therapeutic enhancement. For example, MT destabilization induces an increase in cellular contractility, facilitating improved fluid transport and waste clearance. This mechanism could potentially underlie the results reported in the pilot study by Zinchenko et al., which demonstrated enhanced clearance of amyloid beta in mice treated with transcranial PBM [134]. Additionally, improved fluid flow can positively impact the diffusion and distribution of neurotransmitters, facilitating their delivery to target neurons. PBM-induced cytoskeletal remodeling may also help to restore impaired cell–cell signaling in the NVU.

It is important to recognize that although PBM can modulate cellular processes and affect cellular structures, as demonstrated in this in vitro study, potentially leading to the renewal of microtubules, the outcomes in a living individual with a complex physiological backdrop might be much more variable and unpredictable. An important caveat is that even with high power, NIR light’s penetration into tissues is shallow, often under 2 cm [135], and its effects in deeper regions are largely dependent on indirect signaling pathways and other mechanisms that are not yet fully understood. While positive clinical outcomes have been reported, they stem from small studies. The mechanisms underlying PBM’s impact on tubulin secondary structures, as well as these other influencing factors, still require more comprehensive investigations. The results achieved using an 810 nm wavelength pulsed at 10 Hz are significant; however, exploring different parameters, such as employing a 1060 nm NIR wavelength with a 40 Hz pulse frequency, could yield varied outcomes regarding MT structures. Combined with the current findings, such future studies may enhance the development of more effective PBM devices and treatments. Future clinical studies will be essential to confirm the translational potential of the collective findings.

This study has several limitations, such as the restricted analysis employed and the relatively small number of samples analyzed and experiments performed, which led to sizeable uncertainties in the mean values of secondary structures reported. To address these limitations, replication experiments and analyses of additional Raman modes, such as the amide III band and N–H stretching region, to confirm and further characterize the observed changes in secondary structures and probe the possible breaking of H-bonds are warranted. Supplementing these experimental investigations with in silico studies using molecular dynamics simulations to further validate our results and probe potentially affected residues or domains is also necessary. Furthermore, such studies can provide key insights into the mechanism driving the NIR-induced tubulin conformational changes observed in our experiments.

Additionally, the buffer solution in which the resuspended tubulin was exposed is only an approximation of the intracellular environment. A key difference is that our experiments were conducted with tubulin in the absence of microtubule-associated proteins (MAPs), which appear to play a critical role in AD, especially MAP tau [136,137]. In particular, the inclusion of MAP–tubulin interactions may affect the observed changes in secondary structures induced by NIR radiation (and vice versa), which deserves future study. Moreover, in AD, there appears to be a notable acidification of the intracellular pH (pH_i_) [138,139] associated with a decrease in mitochondrial respiration and connected with a reduction in neuronal activity [140]. Tubulin and MTs are highly sensitive to changes in pH, and the pH of the environment has a significant effect on their behavior and conformation [13,107]. Thus, it would be interesting and valuable to conduct further experiments that investigate how the NIR-induced changes in secondary structures observed in this study might vary as a function of pH. Additionally, the persistence of the conformational changes reported in this study over longer timescales and the potential occurrence of protein refolding also merit further investigation. Lastly, single-cell Raman spectroscopy of NIR-exposed live neuronal cells aimed at studying further possible changes in protein activity at the single neural cell level presents an additional future direction for this research with potential value for the PBM community.

## 5. Conclusions

This study addressed the scarcity of data and conflicting reports in the literature regarding the secondary structures of tubulin in the polymerized state. By employing Raman spectroscopy and subsequent spectral decomposition of the measured amide I spectra of polymerized tubulin samples using a Voigt profile model, this research contributed to our understanding of the conformation of polymerized tubulin. Although spectral deconvolution of Raman spectra based on a Voigt profile has been used previously in several studies to accurately quantify secondary structures of various proteins, this is the first time it has been applied to tubulin or microtubules. Our secondary structure results obtained through Raman spectroscopy confirm the findings of a previous study and help to reconcile the disparities among reported values in the literature. Furthermore, the observed consistency in results obtained served to validate our methodology for this specific context.

Additionally, as the current literature is replete with increasing evidence of the effects of electromagnetic fields on relatively simple structures such as tubulin and microtubules, we sought to investigate potential conformational changes due to exposure to the low-intensity pulsed NIR radiation typically exploited in PBM. Based on our analysis of the irradiated samples, we reported novel findings on the impact of pulsed low-intensity NIR radiation on the secondary structures of polymerized tubulin. We observed a statistically significant decrease in α-helix content and an increase in β-sheets following in vitro exposure to a net dose of 22.5 J/cm^2^ from an 810 nm LED pulsed at a rate of 10 Hz. While there are risks associated with an excessive increase in β-sheets in the context of neurodegenerative diseases due to β-sheet-rich proteins aggregating into amyloids, tubulin has not been connected with amyloid formation. Thus, related clinical evidence supports an interpretation that the PBM-induced conformational changes in tubulins could have a positive impact, leading to refreshed microtubule structures and possibly delaying the aging process.

These structural alterations directly influence the polymerization kinetics of tubulin and microtubules, suggesting potential implications for the efficacy of NIR PBM, particularly when considering potential applications for Alzheimer’s disease and related dementia. Notably, this remodeling appears to offer a refreshment or renewal of microtubules. This study serves not only to bridge key knowledge gaps in the existing literature but also to propose insights into the potential mechanisms by which NIR PBM might be beneficial, especially in the context of exploring therapeutic options for Alzheimer’s disease and other neurodegenerative disorders. Restoring dynamic instability in the dysfunctional microtubules characteristic of NDs could be a pivotal target for PBM therapies. In this regard, this study paves the way for future research to identify the optimal parameters associated with PBM-induced modulation of microtubule dynamics. Further investigations into PBM’s mechanisms are essential to better understand and possibly harness its therapeutic potential for neurodegenerative diseases.

## Figures and Tables

**Table 1:** Characteristic parameters of the intranasal LED applicator of the Vielight Neuro Alpha brain PBM device.

Parameters	Neuro Alpha Intranasal LED
Wavelength (nm)	810
Power density (mW/cm^2^)	25
Pulse frequency (Hz)	10
Pulse duty cycle	50%
Beam spot size (cm^2^)	1

**Table 2:** Secondary structure assignment of amide I band peaks, from Ref. [101].

Secondary Structure	Amide I Band (cm^−1^)
β-sheet	1620–1640, 1670–1680
α-helix	1650–1660
Random coil	1660–1670
β-turn	1680–1699

**Table 3:** Secondary structure composition results obtained for the control (unexposed) polymerized tubulin samples. The last two rows correspond to separate measurements of two different points of the same sample.

Sample	α-Helix	β-Sheet	β-Turn	Random Coil
Control 1	32.7%	26.0%	21.6%	19.7%
Control 2-1	40.7%	19.9%	16.0%	23.5%
Control 2-2	34.5%	34.1%	11.6%	19.8%

**Table 4:** Comparison of the secondary structure percentages for MTs and tubulin (dimeric form).

Material	Source	Method	α-Helix	β-Sheet	β-Turn	Random Coil
MTs	This study (unexposed)	Raman amide I analysis	36.0±4.2%	26.7±7.1%	16.4±5.0%	21.0±2.2%
	Audenaert et al. [13]	Raman amide I analysis	21±3% (**)	48±2% (*)	19±1% (ns)	12±1% (*)
	Simić-Krstić et al. [88]	Raman amide I analysis	33% (ns)	27% (ns)	24% (ns)	16% (ns)
Tubulin	Ventilla et al. [107]	Far-UV CD spectroscopy	22% (*)	30% (ns)		48% (**)
	de Pereda et al. [108]	Far-UV CD spectroscopy	33±7% (ns)	21±5% (ns)	21±6% (ns)	25±6% (ns)
		FTIR spectroscopy	$37^{\;†}\pm 1\%$ (ns)	24±1% (ns)	20±1% (ns)	$18^{\;‡}\pm 1\%$ (ns)
	Afrasiabi et al. [109]	Far-UV CD spectroscopy	38.02% (ns)	15.22% (ns)	–	46.76% (**)

^†^ Maximum. ^‡^ Minimum. ** Highly statistically significant, 0.001≤p<0.01; * statistically significant, 0.01≤p<α; ns, not significant, p≥α.

**Table 5:** Secondary structure composition results obtained for the NIR-exposed polymerized tubulin samples. The last two rows correspond to separate measurements of two different points of the same sample.

Sample	α-Helix	β-Sheet	β-Turn	Random Coil
Exposed 1	15.0%	57.9%	8.7%	18.4%
Exposed 2-1	13.8%	45.5%	24.0%	16.6%
Exposed 2-2	13.0%	59.5%	17.2%	10.3%

**Table 6:** Comparison of secondary structure percentages obtained for control versus NIR-exposed and polymerized tubulin samples. Statistically significant differences between the mean percentages of secondary structures in both groups were determined by Welch’s unequal variances *t*-tests.

Group Analysis	α-Helix	β-Sheet	β-Turn	Random Coil
Control	36.0±4.2%	26.7±7.1%	16.4±5.0%	21.0±2.2%
Exposed tubulin	13.9±1.0%	54.3±7.7%	16.6±7.7%	15.1±4.3%
*p*-value	0.00887	0.0104	0.967	0.123
Significance	**	*	ns	ns

** Highly statistically significant, 0.001≤p<0.01; * statistically significant, 0.01≤p<α; ns, not significant, p≥α.

## Data Availability

The raw data associated with this study are openly available through Figshare at the following DOI: https://doi.org/10.6084/m9.figshare.24492100.

## Supplementary Materials

The following supporting information can be downloaded at: https://www.mdpi.com/article/10.3390/nano14131093/s1.
