# Supplementary material for: Raman Spectroscopy Reveals Photobiomodulation-Induced α-Helix to β-Sheet Transition in Tubulins: Potential Implications for Alzheimer’s and Other Neurodegenerative Diseases
Source: Nanomaterials (Basel). 2024 Jun 26;14(13):1093. doi: 10.3390/nano14131093 (PMC11243591; doi:10.3390/nano14131093)
Supplement: Supplementary file 1 [file nanomaterials-14-01093-s001.zip › nanomaterials-3001315-supplementary.pdf]

Supplementary Materials: Raman Spectroscopy Reveals Photobiomodulation-Induced α-Helix to β-Sheet Transition in Tubulins: Potential Implications for Alzheimer's and Other Neurodegenerative Diseases

Elisabetta Di Gregorio, Michael Staelens, Nazanin Hosseinkhah, Mahroo Karimpoor, Janine Liburd, Lew Lim, Karthik Shankar and Jack A. Tuszyński

S1. Measured Raman Spectra

The first section of this supplementary document presents all of the measured amide I Raman spectra for the experiments described in the main article, along with their corresponding spectral decompositions and R^2 values associated with each fit.

S1.1. Control

The normalized amide I Raman spectra obtained for the control (unexposed) polymerized tubulin samples labeled as Control 1, Control 2-1, and Control 2-2 in Table 3 of the main article are presented in Figures S1–S3, respectively. The results obtained from the deconvolution of each spectrum are overlayed on the plots accordingly.

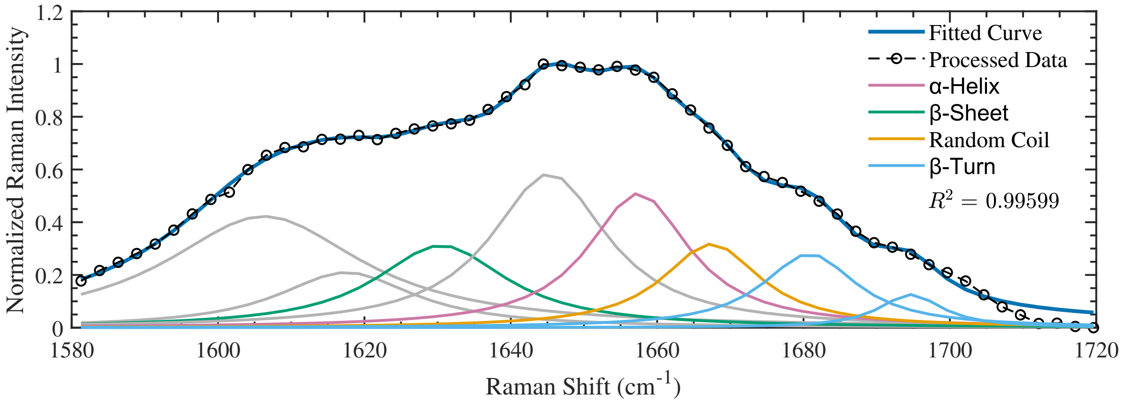

Figure S1. Amide I Raman spectrum obtained for the control (unexposed) polymerized tubulin sample labeled as Control 1 in Table 3 of the main article. Grey curves represent peaks obtained from the spectral deconvolution that are unassociated with any secondary structures.

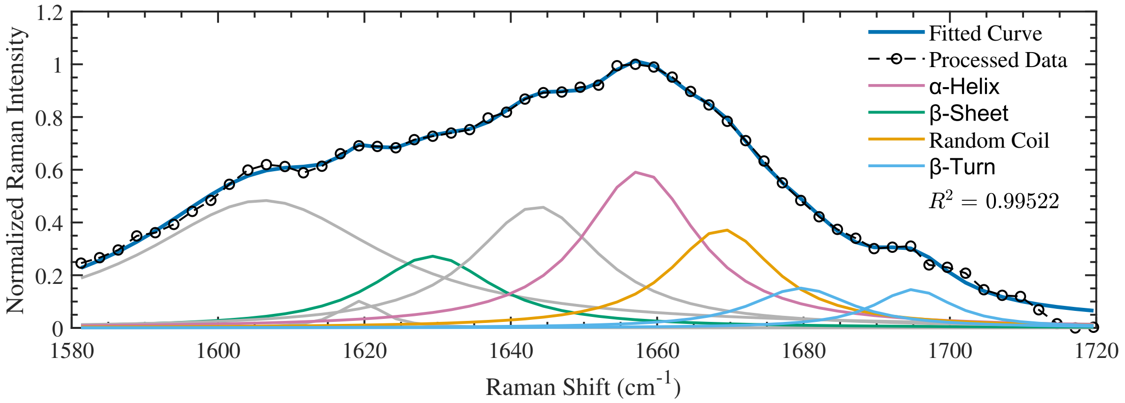

Figure S2. Amide I Raman spectrum obtained for the control (unexposed) polymerized tubulin sample labeled as Control 2-1 in Table 3 of the main article. Grey curves represent peaks obtained from the spectral deconvolution that are unassociated with any secondary structures.

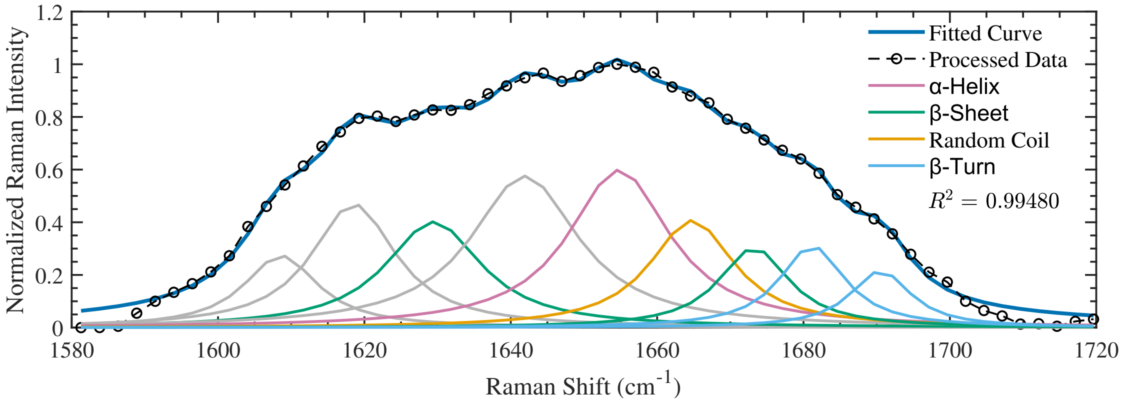

Figure S3. Amide I Raman spectrum obtained for the control (unexposed) polymerized tubulin sample labeled as Control 2-2 in Table 3 of the main article. Grey curves represent peaks obtained from the spectral deconvolution that are unassociated with any secondary structures.

S1.2. NIR-Exposed

The normalized amide I Raman spectra obtained for the NIR-exposed polymerized tubulin samples labeled as Exposed 1, Exposed 2-1, and Exposed 2-2 in Table 5 of the main article are presented in Figures S4–S6, respectively. The results obtained from the deconvolution of each spectrum are overlayed on the plots accordingly.

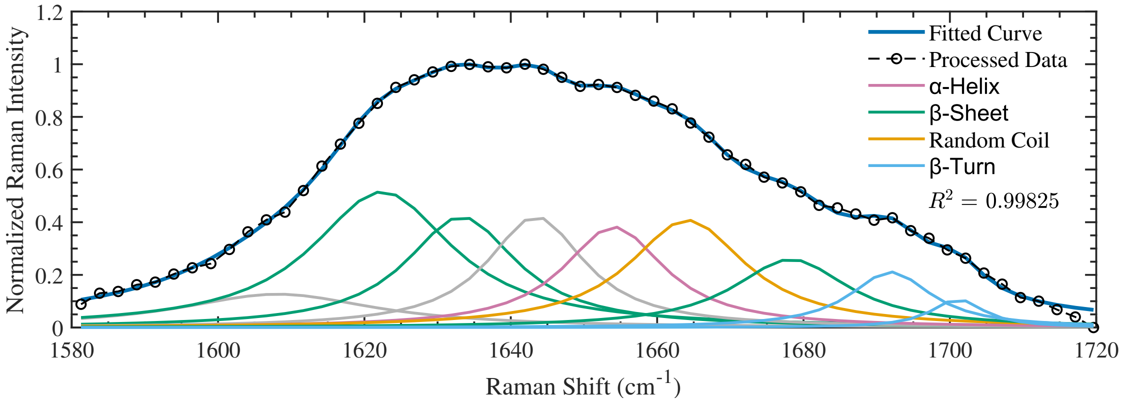

Figure S4. Amide I Raman spectrum obtained for the NIR-exposed polymerized tubulin sample labeled as Exposed 1 in Table 5 of the main article. Grey curves represent peaks obtained from the spectral deconvolution that are unassociated with any secondary structures.

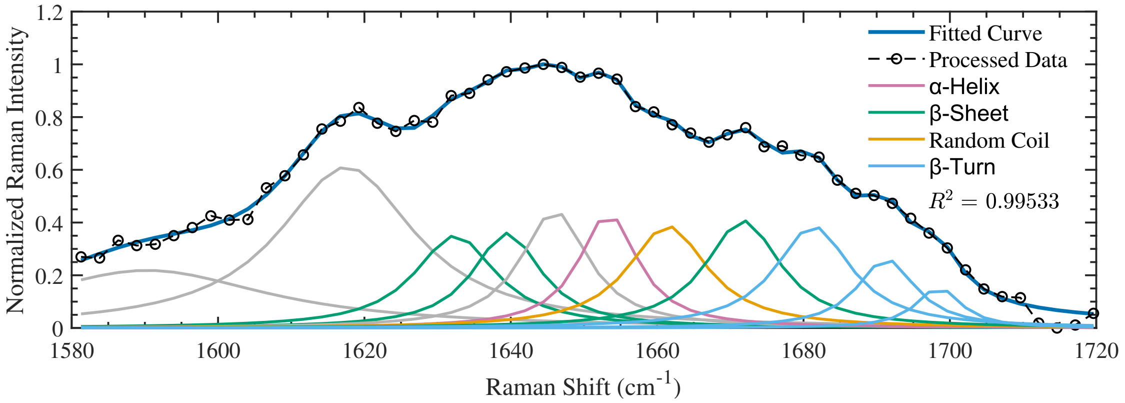

Figure S5. Amide I Raman spectrum obtained for the NIR-exposed polymerized tubulin sample labeled as Exposed 2-1 in Table 5 of the main article. Grey curves represent peaks obtained from the spectral deconvolution that are unassociated with any secondary structures.

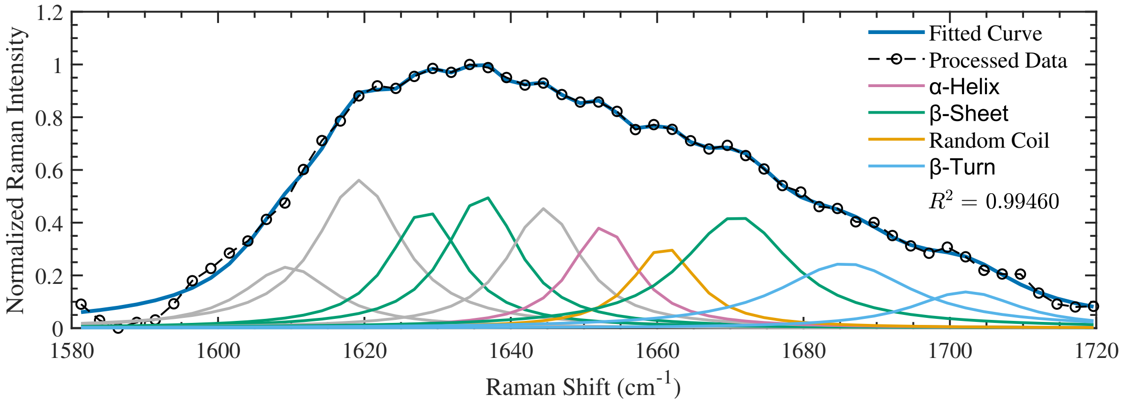

Figure S6. Amide I Raman spectrum obtained for the NIR-exposed polymerized tubulin sample labeled as Exposed 2-2 in Table 5 of the main article. Grey curves represent peaks obtained from the spectral deconvolution that are unassociated with any secondary structures.

S2. Complete Results of Statistical Hypothesis Tests

In the second section of this supplementary document, we report the complete results of all the Welch's unequal variances *t*-tests performed, which were conducted to compare our control sample results with those available in the literature and with the results obtained from our NIR-exposed samples. This choice of statistical hypothesis test was based on unpaired samples, operating under the assumptions of unequal variances and normality. Additional information regarding the experimental methodologies employed by the published studies we compared our results with is also provided here.

S2.1. Control vs. Literature

S2.1.1. MTs vs. MTs

The study conducted by Audenaert et al. [13] utilized Raman spectroscopy and amide I band analysis. Tubulin purified from porcine brain was resuspended in an MES buffer solution (0.1 M, pH 6.4) containing 50 mM MES, 1 mM EGTA, 1 mM $MgCl_2$, 1 mM NaN_3, and 70 mM NaCl to a protein concentration ranging from 40 to 50 mg/ml. To facilitate polymerization, 1 mM GTP and 1 mM Mg^{2+} were added to the solution, and the glycerol content was increased to 4 M. Tubulin was then polymerized into microtubules at 37 °C for 30 min. Raman spectroscopy measurements were carried out at 25 °C using a 514 nm Ar^+ laser with a power of 100 mW and a 5 s integration time. The study involved at least four different samples; standard deviations in the secondary structures were 3% for the ordered

helix, 2% for the antiparallel beta-sheet, and 1% for the other structural classes. The full results of Welch's unequal variances *t*-tests comparing our secondary structure values for polymerized tubulin versus their values are reported in Table S1.

Table S1. The results of Welch's *t*-tests comparing the secondary structure results obtained for the unexposed polymerized tubulin samples in our study vs. the results reported by Audenaert et al. [13] for polymerized tubulin (using Raman spectroscopy). DF represents the degrees of freedom, and h_{null} represents the test decision regarding the rejection of the null hypothesis, such that a value of 1 indicates a rejection of the null hypothesis at the 5% significance level.

Secondary Structure	*t*-value	DF	h_{null}	*p*-value
α-Helix	5.25	3.49	1	0.00913
β-Sheet	−5.04	2.24	1	0.0294
β-Turn	−0.885	2.12	0	0.465
Random Coil	6.68	2.65	1	0.0100

Simić-Krstić et al. [88] also employed amide I band analysis of Raman spectra. In their study, tubulin protein sourced from pig brain was resuspended in a buffer solution with a pH of 6.5 and subsequently polymerized into microtubules at 35 °C. Raman spectroscopy measurements were conducted at a temperature of 22 ± 2 °C using a 514.5 nm Ar^+ laser and an integration time of 3 s. It is worth noting that the study did not report errors or standard deviations in the results, and the precise number of samples analyzed and experiments conducted was not specified. The full results of Welch's unequal variances *t*-tests comparing our secondary structure values for polymerized tubulin versus their values are reported in Table S2.

Table S2. The results of Welch's *t*-tests comparing the secondary structure results obtained for the unexposed polymerized tubulin samples in our study vs. the results reported by Simić-Krstić et al. [88] for polymerized tubulin (using Raman spectroscopy). DF represents the degrees of freedom, and h_{null} represents the test decision regarding the rejection of the null hypothesis, such that a value of 1 indicates a rejection of the null hypothesis at the 5% significance level.

Secondary Structure	*t*-value	DF	h_{null}	*p*-value
α-Helix	1.22	2	0	0.345
β-Sheet	−0.0810	2	0	0.943
β-Turn	−2.63	2	0	0.120
Random Coil	4.00	2	0	0.0572

S2.1.2. MTs vs. Tubulin

Ventilla et al. [107] employed far-UV circular dichroism (CD) spectroscopy to investigate the secondary structure of tubulin protein purified from pig brain. Based on the measured CD spectra, secondary structures were quantified by applying fits to standard curves for α-helix, β-structures, and random coil with a corresponding precision of approximately 1%. The tubulin protein was prepared at 4 °C in phosphate buffer with a pH of 6.5, containing 0.05 M phosphate, 10^{-2} M Mg^{2+}, and 10^{-3} M GTP. Samples of around 0.6 ml with a protein concentration of approximately 0.1 mg/ml were used in their measurements. The CD spectra of isolated tubulin dimers were obtained at various pH values and two temperatures (4 °C and 37 °C), with a digitization interval of 1.0 nm. We compared with their results for pH 6.5 and 4 °C (for which the authors claim no significant turbidity-based artifacts affecting the results). Additionally, their quantification of secondary structures did not distinguish between β-sheets and β-turns; thus, in our comparisons with their results, we combined our β-sheet and β-turn values. The specific number of samples analyzed and the total count of independent experiments performed were not reported, nor was the accuracy in their CD-based secondary structure quantification results explicitly stated in

the study. The full results of Welch's unequal variances *t*-tests comparing our secondary structure values for polymerized tubulin versus their values are reported in Table S3.

Table S3. The results of Welch's *t*-tests comparing the secondary structure results obtained for the unexposed polymerized tubulin samples in our study vs. the results reported by Ventilla et al. [107] for dimeric tubulin (using far-UV CD spectroscopy). DF represents the degrees of freedom, and h_{null} represents the test decision regarding the rejection of the null hypothesis, such that a value of 1 indicates a rejection of the null hypothesis at the 5% significance level.

Secondary Structure	*t*-value	DF	h_{null}	*p*-value
α-Helix	5.76	2	1	0.0288
β-Structures	2.60	2	0	0.122
Random Coil	−21.6	2	1	0.00214

De Pereda et al. [108] quantified the secondary structures of dimeric tubulin protein using two different methods: far-UV CD spectroscopy and Fourier-transform infrared spectroscopy (FTIR) spectroscopy. The tubulin used in their experiments was purified from calf brain. For the far-UV CD measurements, they employed two different buffers: 1) a 10 mM sodium phosphate buffer at pH 7.0 and 2) a 10 mM sodium phosphate buffer supplemented with 6 mM $MgCl_2$ at pH 6.7. Tubulin was resuspended in these buffers in the presence of 0.1 mM GTP to a final protein concentration of 20 μM. Four independent samples were measured—two samples for each buffer solution. The far-UV CD measurements were performed at 25 °C with a 1 nm bandwidth, acquiring four scans between 185 and 260 nm, which were subsequently averaged. The resulting far-UV CD spectra were analyzed using six different methods. Individual results for the secondary structure composition of tubulin determined by each method were reported, along with the results obtained by averaging over all methods. We performed our comparisons with their averaged results, which included clearly stated standard deviations for each result.

In their FTIR measurements, amide I′ spectra of tubulin resuspended in sodium phosphate and D_2O buffer (10 mM sodium phosphate, 0.1 mM GTP, pH 7.4) were obtained at 25 °C. Samples with final protein concentrations of 20 and 50 μM were analyzed. Spectra were acquired over 1000–2000 cm^{-1} at a 2 cm^{-1} resolution. Background subtractions were applied to account for the absorbances of atmospheric water vapor and the buffer solution. Results reported were based on the average of two independent measurements performed with different instruments; a maximum standard deviation of 1% was stated, which we used in our comparisons with their FTIR results. The full results of Welch's unequal variances *t*-tests comparing our secondary structure values for polymerized tubulin versus their results obtained using far-UV CD and FTIR spectroscopy are reported in Tables S4 and S5, respectively.

Table S4. The results of Welch's *t*-tests comparing the secondary structure results obtained for the unexposed polymerized tubulin samples in our study vs. the results reported by de Pereda et al. [108] for dimeric tubulin (using far-UV CD spectroscopy). DF represents the degrees of freedom, and h_{null} represents the test decision regarding the rejection of the null hypothesis, such that a value of 1 indicates a rejection of the null hypothesis at the 5% significance level.

Secondary Structure	*t*-value	DF	h_{null}	*p*-value
α-Helix	0.697	4.88	0	0.518
β-Sheet	1.18	3.44	0	0.314
β-Turn	−1.10	4.86	0	0.321
Random Coil	−1.23	3.95	0	0.287

Afrasiabi et al. [109] conducted a far-UV CD spectroscopic analysis of the conformation of dimeric tubulin purified from sheep brain (purity > 95%). The tubulin was resuspended in PEM buffer (comprising 100 mM PIPES at pH 6.9, 2 mM $MgSO_4$, and 1 mM EGTA) to a

Table S5. The results of Welch's t-tests comparing the secondary structure results obtained for the unexposed polymerized tubulin samples in our study vs. the results reported by de Pereda et al. [108] for dimeric tubulin (using FTIR spectroscopy). DF represents the degrees of freedom, and h_{null} represents the test decision regarding the rejection of the null hypothesis, such that a value of 1 indicates a rejection of the null hypothesis at the 5% significance level.

Secondary Structure	t-value	DF	h_{null}	p-value
α-Helix	−0.409	2.32	0	0.717
β-Sheet	0.639	2.12	0	0.585
β-Turn	−1.21	2.23	0	0.339
Random Coil	2.09	2.89	0	0.131

final protein concentration of 2 mg/ml. CD spectra of the tubulin dimer solution were measured in the 190–260 nm range; the measurement temperature was not explicitly mentioned. Deconvolution of the resulting CD spectra was performed to determine the secondary structure content. The exact quantity of samples analyzed and the total number of independent experiments performed were not reported, nor were any uncertainties provided for the resulting tubulin secondary structure values. The full results of Welch's unequal variances t-tests comparing our secondary structure values for polymerized tubulin versus their values are reported in Table S6.

Table S6. The results of Welch's t-tests comparing the secondary structure results obtained for the unexposed polymerized tubulin samples in our study vs. the results reported by Afrasiabi et al. [109] for dimeric tubulin (using far-UV CD spectroscopy). DF represents the degrees of freedom, and h_{null} represents the test decision regarding the rejection of the null hypothesis, such that a value of 1 indicates a rejection of the null hypothesis at the 5% significance level.

Secondary Structure	t-value	DF	h_{null}	p-value
α-Helix	−0.847	2	0	0.486
β-Sheet	2.78	2	0	0.108
Random Coil	−20.6	2	1	0.00235

A general overview of some of the key differences in the sample and experimental conditions between the studies compared is provided in Table S7.

S2.2. Exposed vs. Control

The complete results of Welch's unequal variances t-tests comparing our secondary structure values obtained for unexposed vs. NIR-exposed polymerized tubulin samples are reported in Table S8.

Table S7. Comparative overview of the sample and experimental conditions across studies on tubulin secondary structures in the polymerized and unpolymerized states.

Sample	Study	Sample Source	Buffer	pH	$C_{\mathbf{sample}}$	$T_{\mathbf{m}}$
MTs	This study	Porcine Brain	PEM	6.9	2.5 mg/ml	22 °C
	Audenaert et al. [13]	Porcine Brain	MES	6.4	40–50 mg/ml	25 °C
	Simić-Krstić et al. [88]	Porcine Brain	N/A	6.5	N/A	22 ± 2 °C
Tubulin	Ventilla et al. [107]	Porcine Brain	Phosphate	6.5	0.1 mg/ml	4 °C
	de Pereda et al. (far-UV CD) [108]	Calf Brain	Sodium Phosphate	7.0	2.2 mg/ml	25 °C
	de Pereda et al.(FTIR) [108]	Calf Brain	Sodium Phosphate	7.4	2.2 and 5.5 mg/ml	25 °C
	Afrasiabi et al. [109]	Ovine Brain	PEM	6.9	2 mg/ml	N/A

N/A: information not available.

Table S8. The full results of Welch's t-tests comparing the secondary structure results obtained in our study for the unexposed polymerized tubulin samples vs. the NIR-exposed polymerized tubulin samples. DF represents the degrees of freedom, and h_{null} represents the test decision regarding the rejection of the null hypothesis, such that a value of 1 indicates a rejection of the null hypothesis at the 5% significance level.

Secondary Structure	t-value	DF	h_{null}	p-value	|Lower CI|	|Upper CI|
α-Helix	8.84	2.23	1	0.00887	12.3	31.8
β-Sheet	−4.57	3.98	1	0.0104	10.8	44.4
β-Turn	−0.0441	3.45	0	0.967	15.4	15.9
Random Coil	2.14	2.97	0	0.123	2.92	14.7
